# SPIN-AI: A Deep Learning Model That Identifies Spatially Predictive Genes

**DOI:** 10.3390/biom13060895

**Published:** 2023-05-27

**Authors:** Kevin Meng-Lin, Choong-Yong Ung, Cheng Zhang, Taylor M. Weiskittel, Philip Wisniewski, Zhuofei Zhang, Shyang-Hong Tan, Kok-Siong Yeo, Shizhen Zhu, Cristina Correia, Hu Li

**Affiliations:** 1 Department of Molecular Pharmacology and Experimental Therapeutics, Mayo Clinic, Rochester, MN 55905, USA; meng-lin.kevin@mayo.edu (K.M.-L.); ung.choongyong@mayo.edu (C.-Y.U.); zhang.cheng@mayo.edu (C.Z.); weiskittel.taylor@mayo.edu (T.M.W.); wisniewski.philip@mayo.edu (P.W.); zhuofei.zhang20@student.xjtlu.edu.cn (Z.Z.); tan.shyanghong@mayo.edu (S.-H.T.); 2 Department of Biochemistry and Molecular Biology, Mayo Clinic College of Medicine and Science, Rochester, MN 55905, USA; yeo.koksiong@mayo.edu (K.-S.Y.); zhu.shizhen@mayo.edu (S.Z.)

**Keywords:** spatial transcriptomics, artificial intelligence, spatial gene regulation, cellular niche

## Abstract

Spatially resolved sequencing technologies help us dissect how cells are organized in space. Several available computational approaches focus on the identification of spatially variable genes (SVGs), genes whose expression patterns vary in space. The detection of SVGs is analogous to the identification of differentially expressed genes and permits us to understand how genes and associated molecular processes are spatially distributed within cellular niches. However, the expression activities of SVGs fail to encode all information inherent in the spatial distribution of cells. Here, we devised a deep learning model, Spatially Informed Artificial Intelligence (SPIN-AI), to identify spatially predictive genes (SPGs), whose expression can predict how cells are organized in space. We used SPIN-AI on spatial transcriptomic data from squamous cell carcinoma (SCC) as a proof of concept. Our results demonstrate that SPGs not only recapitulate the biology of SCC but also identify genes distinct from SVGs. Moreover, we found a substantial number of ribosomal genes that were SPGs but not SVGs. Since SPGs possess the capability to predict spatial cellular organization, we reason that SPGs capture more biologically relevant information for a given cellular niche than SVGs. Thus, SPIN-AI has broad applications for detecting SPGs and uncovering which biological processes play important roles in governing cellular organization.

## 1. Introduction

The burgeoning of single-cell sequencing technologies over the past decade [1] has revolutionized our understanding of many important biological problems at single-cell resolution. Among them are issues pertaining to cellular heterogeneity [2], cell subpopulation [3], and fate determination of cell lineages [4] during embryonic development and disease formation [5,6]. However, the process of dissociating cells from their tissue before sequencing causes the loss of spatial information in single-cell RNA-seq data.

The coupling of novel next-generation sequencing-based [5,7,8] and imaging-based capturing [9,10,11] spatial-based approaches over the last few years has inhibited the loss of spatial information in cell sequencing. Spatially resolved transcriptomics has since enabled researchers to chart how gene expression varies across different regions of a tissue in unprecedented detail [12]. Although different spatial sequencing technologies have their own limitations, such as resolution, sensitivity, throughput, and accessibility [13], the availability of spatial transcriptomic data nonetheless allows researchers to address long-awaited, important biological questions. For instance, spatial transcriptomic data have allowed researchers to build detailed tissue expression atlases [14], trace tissue development at refined spatial resolutions [15] and dissect cell–cell communication patterns in cellular niches [16].

More importantly, the advancement of spatial sequencing technologies has also uncovered new computational challenges in revealing how the spatial arrangement of cells affects gene expression or vice versa. That is, a cell’s local neighborhood in space may determine how the cell expresses its genes, and the coordinated gene expression of cells in a neighborhood shape the property of the niche in which they reside. As such, reconstructing spatial positions and inferring genes whose expression patterns play key roles in molding spatial organization are attractive computational problems.

Restoring lost spatial information from single-cell sequencing data via the in silico reconstruction of spatial data has been an active research area in recent years. For example, novoSpaRc was developed to reconstruct de novo cellular spatial locations of cells using single-cell transcriptomic data without the need for spatial references [14]. The authors’ key assumption was that physically adjacent cells tend to share similar gene expression profiles, and spatial position probabilities for individual cells can be formulated as optimal transportation problems [17]. On the other hand, CSOmap was built using information on ligand–receptor interactions to reconstruct cellular spatial organization from single-cell RNA-seq data [11]. The underlying hypothesis of CSOmap is that the spatial organization of cells is determined by their local ligand–receptor interaction profiles. By reconstructing spatial organization, both methods also infer spatially informative expression programs. The success of both novoSpaRc and CSOmap in reconstructing the spatial organization of single-cell data, at least to a certain extent, indicates that gene expression profiles encode the latent representations of cells in space.

Beyond reconstructing spatial information from single-cell RNA-sequencing, another line of effort has employed spatially resolved transcriptomic data to explore the spatial organization of gene expression. This includes the identification of spatially variable genes (SVGs), whose expression patterns are significantly distinct across space. That is, the expression of SVGs is highly localized to specific zones and demonstrates a spatial pattern, whereas non-spatially variable genes are expressed in broad locations within cellular niches. As such, most current computational methods for detecting SVGs are mainly based on statistical hypothesis-testing frameworks, such as trendsceek, which uses spatial point process models [18], or SpatialDE [19] and SPARK [20], which use Gaussian processes. Hybrid methods that combine machine learning and statistical methods have also gained interest, such as SOMDE, which combines self-organizing maps and Gaussian process models to detect SVGs [21].

Both de novo computational methods for reconstructing spatial organization from single-cell RNA-seq data (e.g., novoSpaRc and CSOmap) and statistical methods for detecting SVGs (e.g., trendsceek, SpatialDE, SPARK, and SOMDE) assume that gene expression (or interaction) programs that show similarities at adjacent localities are the most spatially informative. However, expression in a spatial-dependent manner does not necessarily imply that only SVGs are involved in the gene expression behaviors that coordinate the spatial organization of cells. SVGs and non-SVGs may interact together to encode spatial information. As such, we predict the existence of a new class of genes called spatially predictive genes (SPGs). Although SPGs are not necessarily spatially variable, their collective expression levels nonetheless encode information for reconstructing the organization of cells in space. The concept of an SPG, therefore, merges two different aspects of single-cell analyses, i.e., the reconstruction of the spatial organization of cells and the detection of spatially expressed genes, into one umbrella: genes whose expression activities predict the coordinated distribution of cells in space.

In this study, we developed a deep learning computational pipeline called Spatially Informed Artificial Intelligence (SPIN-AI) to identify SPGs without any prior assumptions of spatial distribution. We used data on human squamous cell carcinoma from a study by Ji et al. [22] in our case studies. SPIN-AI learns to reconstruct the spatial transcriptomic slide of a cell using only its gene expression profiles by identifying SPGs during its training process. We hypothesize that some SPGs are potential players involved in spatial-based regulatory mechanisms that determine and coordinate cellular distribution in the pathological microenvironment. Our work shows that many SPGs are not SVGs, indicating that SPGs are a new class of genes whose collective expression profiles encode the spatial distribution of cells in a cellular niche. We also showed that SPG-enriched biological processes recapitulate the biological properties of squamous cell carcinoma. Hence, SPIN-AI predicted that patient-specific SPGs will be of great value in understanding the coordination of the gene activities that give rise to the spatial organization of cells in a pathological microenvironment.

## 2. Materials and Methods

### 2.1. Packages

For this study, we used the following Seurat [23], STUtility, clusterProfiler, ggplot2, data.table, spatialDE [19], ComplexHeatmap, circlize, tibble, openxlsx, VennDiagram, and Python 3 packages: numpy, pandas, tensorflow, keras, deepexplain, seaborn, matplotlib, itertools, sys, os, gc, and re. Information about the use of each package is listed in Table S1 and Figure S1.

### 2.2. Data Pre-Processing

Human squamous cell carcinoma single-cell and spatial transcriptomic data were obtained from Ji et al. [22] The data consisted of matched normal and tumor single-cell RNA-seq as well as 10× Visium spatial transcriptomic profiles with matched histology stains for 3 tumor tissue sections across 4 patients. We henceforth refer to the matched spatial transcriptomic and histology stains as “slides,” with a total of 12 slides, or 3 for each of the 4 patients. Both single-cell and spatial transcriptomic data were processed using Seurat [21] and STutility [24], as described in Ji et al. [22] Briefly, for the single-cell data, cells with <200 genes detected and >10% mitochondrial gene counts were filtered out. The data were then normalized and scaled, while UMI counts and mitochondrial gene percentages were regressed out. For the spatial transcriptomic data, spots with <200 genes detected were removed as were genes with <10 reads or expression in <2 spots. Data were then normalized (SCTransform function), while slide-specific effects and gene counts per spot were regressed out.

### 2.3. Cellular Subpopulation and Cluster Analysis

We obtained single-cell identity annotations and computed spatial gene expression clusters by following the procedure described by Ji et al. [22]. This information was used for comparison against post-hoc models and was not included in our deep learning models. From the single-cell RNA-seq data, we used pre-generated cell-type labels to conduct a Bonferroni-corrected Fisher’s exact test to compare the cell-type proportions in tumor samples from Patient 9 against those from Patients 2, 5, and 10 aggregated together, since the model performance for Patient 9 was significantly reduced compared to these patients (see Results Section). We then computed gene expression markers for spatial expression clusters using Seurat’s FindAllMarkers function [23]. We computed another set of markers using gene importance scores (as calculated in “Identification of Spatially Predictive Genes (SPGs)”) for comparison against the expression-derived markers. Finally, we extracted the 200-gene signature for tumor-specific keratinocytes (TSKs) identified in Ji et al., generated TSK scores, and identified the spatial expression clusters enriched for these cells using Seurat’s AddModuleScore function [23].

### 2.4. SPIN-AI Training Procedure

**Figure 1:**
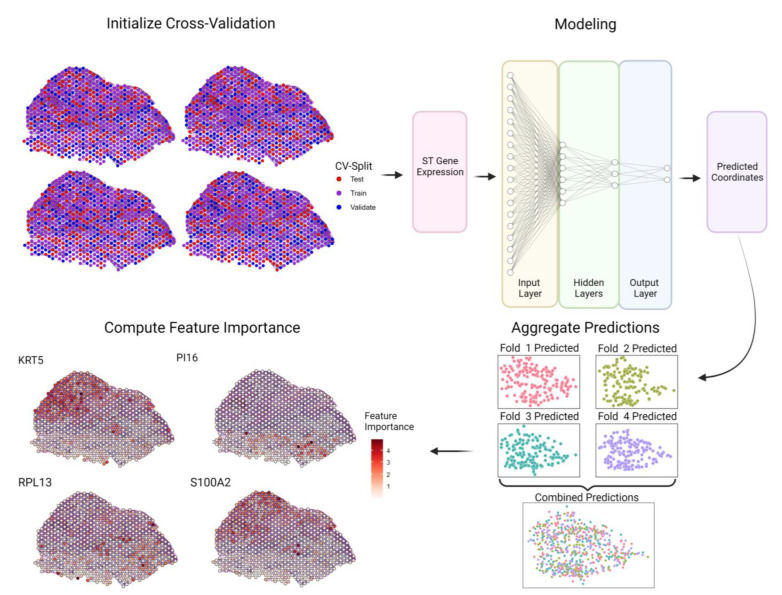
**Design of the Spatially Informed Artificial Intelligence (SPIN-AI) platform.** SPIN-AI consists of a dense, feedforward neural network where spatial transcriptomic gene expression is used as an input with the goal of predicting the *x* and *y* coordinates of each spatial transcriptomic spot. Each hidden layer of the model consists of a dense, fully connected layer. The number of hidden layers was tuned between 1, 3, and 5. The number of nodes in the hidden layer was fixed at half the input layer size and distributed such that each hidden layer had half of the previous layers’ number of nodes. For a given slide, spots are randomized to different folds for k-fold cross-validation (k = 4 shown for illustration purposes). A deep, feedforward neural network is then trained on the training folds to predict spatial location from spatial gene expression and evaluated according to its predictions for spots from the test fold. Test fold predictions are then aggregated for model evaluation and feature importance is computed for each gene for each spot. Each dotted spot represents a spot on the spatial transcriptomic slide.

SPIN-AI consists of a dense, feedforward neural network designed to take a spatial transcriptomic spot’s gene expression as an input, with each node corresponding to one gene, to predict its *x* and *y* spatial coordinates (Figure 1). Prior to training, genes with an expression variance of <0.05 were filtered out. Each hidden layer of a model consists of a dense, fully connected layer followed by batch normalization. The number of hidden layers was tuned between 1, 3, and 5 per patient, as described below. The total number of nodes in the hidden, dense layers was fixed at approximately half of the input layer size, and the distribution of nodes in a model was fixed at half the number of nodes from the preceding layer, except for in the 2-node output layer for predicting *x* and *y* coordinates. For all dense layers, we used a rectified linear unit (ReLu) activation function with He normal weight initialization [25] and an Adam optimization function [26]. The ReLu activation function is given as followed:

(1) $$ReLu\left(x\right)=max(0,x),$$

where x is the input to a neuron.

Each SPIN-AI model was built on a per-slide basis. The learning rate was tuned between 0.1, 0.01, and 0.001 using 10-fold cross-validation. In each cross-validation iteration, 8 folds were used for training, 1 was selected for validation, and another was used for testing. We trained each model using the mean Euclidean distance error (MDE) as a loss function, measuring the average distance from each spot’s predicted location to their actual location:

(2) $$MDE=\frac{1}{N}\sum_{n}^{N}\sqrt{{\left(x_{n}^{\prime }-x_{n}\right)}^{2}+{\left(y_{n}^{\prime }-y_{n}\right)}^{2}},$$,

where *N* is the number of spots, $\left(x_{n},y_{n}\right)$ is a spot’s coordinate, and ($x_{n}^{\prime },y_{n,}^{\prime }$) is the predicted coordinate for spot *n*.

To prevent overfitting, we used early stopping within each fold alongside a batch size of 32 and 100 epochs. After cross-validation, we selected the parameter combination with the lowest cross-validation error and selected its corresponding set of within-fold models. We collected the predictions for each models’ test fold for subsequent analysis and computed gene contribution scores (detailed below) for the test-fold spots using their respective models.

### 2.5. Model Validation

First, we verified that the cross-validation error could approximate the actual error by using all three slides from each patient to train the models. Using slides from the same patient, we trained each model on one selected slide with optimal hyperparameters determined from the cross-validation procedure performed on that slide. Another slide was used as a validation set with the remaining slide used as a test set. To ensure consistency between each slide’s coordinates, we computed the centers of each slide by taking their average *x* and *y* coordinates. We then computed the difference between the testing and validation slides’ centers and the training slide center, and then translated the testing and validation slides such that their translated image centers had the same coordinates as the training slide. The train–validation–test slides were tested in the following combinations: Slide 1, Slide 2, and Slide 3; Slide 2, Slide 3, and Slide 1; and Slide 3, Slide 1, and Slide 2.

### 2.6. Identification of Spatially Predictive Genes (SPGs)

We used DeepLift [27], as implemented in the DeepExplain library, to compute spot-wise node contribution scores. For a specific spot *s*, outcome *t,* and nodes $x_{1}^{s},\;x_{2}^{s},\ldots ,x_{n}^{s}$ where *n* is the number of nodes in a given layer *l*, DeepLift calculates the contribution $C_{i}^{s,l}$ for each node i∈n subject to the constraint that

(3) $$\sum_{\;i=1}^{\;n}C_{i}^{s,l}=t-t_{0},$$

where $t_{0}$ is the model output when all nodes in layer *l* are activated with a certain reference input, which was 0 for our scenario. In essence, DeepLift calculates how much a given node contributed to a prediction.

Within an individual cross-validation iteration, we applied DeepLift to the input layer of the trained model to compute how much each gene contributed to the prediction of the location of each spot in the test set. From the cross-validation design, we then obtained the gene contribution scores of all genes in all spots in a slide $C_{g}^{s}$, where g denotes the gene and s denotes the spot. We henceforth refer to the magnitude of the gene contribution scores as the “importance score,” or

(4) $${Imp}_{g}^{s}=\left|C_{g}^{s}\right|.$$

We evaluated gene importance across an entire slide by taking the sum of importance scores as

(5) $${MeanImp}_{g}=\frac{1}{\left|S\right|}\sum_{s}^{S}{Imp}_{g}^{s}$$

where *S* is the set of all spots in a slide.

We then determined SPGs on a per-patient basis (Figure S2). First, we computed the cross-slide mean importance (CSMI score) of each gene across each of the three slides for a patient:

(6) $${CSMI}_{g}=\frac{1}{\left|H\right|}\sum_{h}^{H}{MeanImp}_{g}^{h},$$

where *h* denotes each individual slide in the set of all slides *H* (*|H|* = 3 in this case).

Because MeanImp and, thus, CSMI are correlated with the average expression in a slide/across slides (Figure S3), MeanImp may deprioritize genes with strong regional bias. To account for genes with strong contribution scores in a subset of spots, we computed the mean non-zero importance (MNI) for each gene on a per-slide basis as

(7) $${MNI}_{g}=\frac{1}{n_{0}}\sum_{s}^{S}{Imp}_{g}^{s},$$

where $n_{0}$ is the number of spots with ${Imp}_{g}^{s}$ > 0. We also computed the percentage of spots with non-zero importance (PNI) as

(8) $${PNI}_{g}=100*\frac{n_{0}}{n}$$

As with the CSMI, we calculated the cross-slide averaged MNI (CSMNI) and the cross-slide averaged PNI (CSPNI) for each gene across all three patient slides:

(9) $$CSMNI=\frac{1}{\left|H\right|}\sum_{h}^{H}{MNI}_{g}^{h}\;\mathrm{and}\;CSPNI=\frac{1}{\left|H\right|}\sum_{h}^{H}{PNI}_{g}^{h}.$$

To define a set of SPGs, we initially selected genes that had a CSMI > 0.15. Our choice of CSMI cutoff was based on scores of 0.15 falling in between the 98th and 99th percentile of importance scores across all genes and slides for each patient (98.47th percentile for Patient 2, 99.22nd percentile for Patient 5, 98.25th percentile for Patient 9, 98.48th percentile for Patient 10), providing a sufficiently selective cutoff. We then computed the minimum CSMNI for genes in this initial set. Next, we selected genes with a CSMNI value and a CSPNI > 20 (Figure S2) to help detect genes with strong importance in a reasonably sized subset of spots. Adding these genes to the initial gene set formed the spatially predictive genes (SPGs) of a patient. We compared the impacts between using means or medians for equations 5, 6, 7, and 9. Since the Pearson correlation coefficients between the two aggregations methods were strong (>0.9), we reasoned that no significant changes would be expected when using either scoring scheme and, thus, used the mean for our calculations (Figure S4). On a per-patient basis, we also used SpatialDE [19] to detect spatially variable genes using an adjusted *p*-value threshold of 0.05.

### 2.7. Enrichment Analyses

To help interpret the gene importance scores and functionality of identified SPGs, we conducted Gene Ontology overrepresentation analysis on spatially predictive features (SPGs) using the biological process gene sets annotated in Gene Ontology (GO) with a Benjamini–Hochberg corrected *p*-value < 0.05. A similar procedure was performed using molecular-function gene sets to compare cluster markers and biological-process gene sets to compare SPGs and SVGs.

## 3. Results

### 3.1. Design of the Spatially Informed Artificial Intelligence (SPIN-AI) Platform

The underlying hypothesis for the design of SPIN-AI is that there exists a certain class of genes, namely spatially predictive genes (SPGs), whose collective expression activities encode information dictating the spatial distribution of cells in a given cellular niche. This is unlike spatially variable genes (SVGs), which show differential expression across space; SPGs themselves may not show considerable spatial variation, i.e., they can express throughout cellular niches, but their collective expression profiles encode spatial positioning. SPGs may also include SVGs and, hence, capture a broader set of genes than SVGs [19].

Here, we designed SPIN-AI using a dense, feedforward deep neural network as a computational framework for learning SPGs (Figure 1). As neural networks can accurately model linear and non-linear systems, we reason that neural networks can learn SPGs via a supervised learning process [28]. SPIN-AI takes gene expression per spot as an input and is then trained to predict the *x* and *y* spatial coordinates of a spatial transcriptomic slide. The learning rate hyperparameter and the number of hidden layers were tuned per patient, as described in the methods. To assess model performance, spots were randomized to different folds for 10-fold cross-validation. Test fold predictions were then aggregated for model evaluation and used to create a predicted spatial distribution. The contribution of each gene’s expression for predicting the spatial distribution of spots was scored according to feature importance and used to derive SPGs (Figure S2).

### 3.2. Trained SPIN-AI Models Can Predict Spatial Organization of Spots on Slide Per Patient

**Figure 2:**
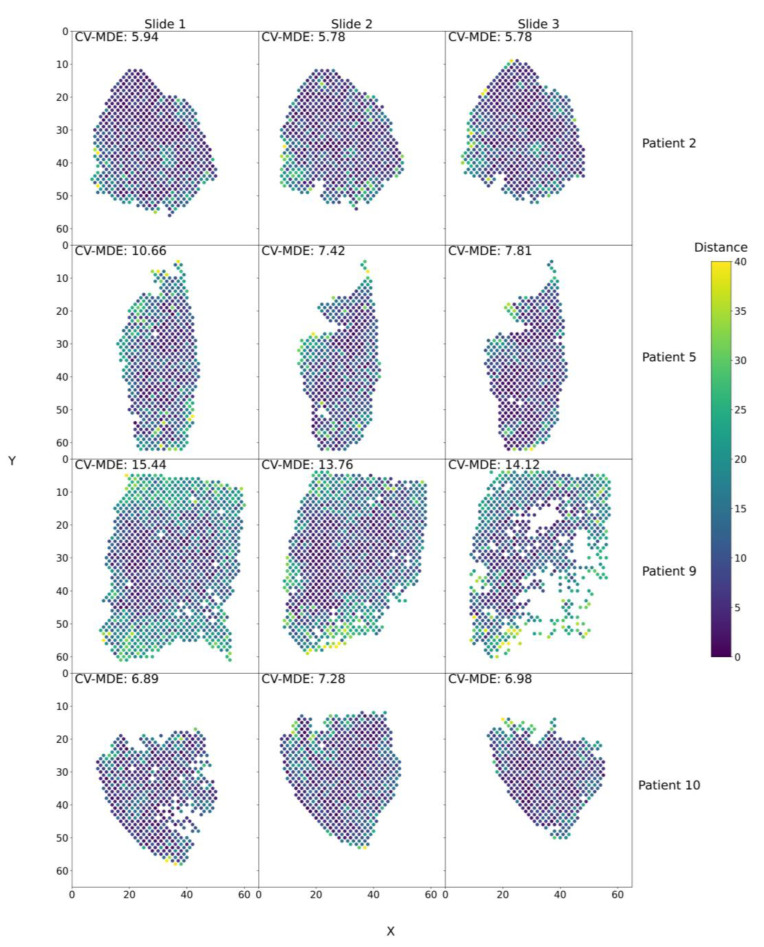
**SPIN-AI Model performance for all patients and slides.** Spatial transcriptomic spots are arranged in their original positions and colored according to the distance from their predicted locations to their actual locations. The MDE, averaged across CV folds (CV-MDE), is shown in the top left corner. The model’s performance was evaluated using the best hyperparameter combination per patient. For Patients 2, 5, and 10, the best models were those with five hidden layers and a learning rate of 0.001. For Patient 9, the best model was the model with three hidden layers and a learning rate of 0.001. Spots are approximately two coordinate units apart.

We tested our modeling approach on a dataset of four squamous cell carcinoma patients from Ji et al. [22] Each patient had three 10× Visium spatial transcriptomic slides taken from their tumors. SPIN-AI models were trained and fine-tuned with respect to each patient and slide, as the best model structure may vary. The best models across all slides for Patients 2, 5, and 10 were the models with five hidden layers and a learning rate of 0.001. The best model across all slides for Patient 9, was a model with three hidden layers and a learning rate of 0.001. We tested the performance of these models for their capability to predict spots in their respective locations on a slide per patient. The predicted *x* and *y* coordinates for a given spot are assessed according to its actual *x* and *y* coordinates using the mean distance error (MDE). Figure 2 shows that our trained models performed reasonably well in predicting the *x* and *y* coordinates of spots on a slide, except for in Patient 9, who showed a poorer performance than the others due to an increased presence of migratory immune cells (T-cells, macrophages, dendritic cells). In addition, the models also showed consistent performance for all three slides per patient, further indicating the models’ ability to capture biological signals (Figure S5). The capability of trained SPIN-AI models to predict spot locations on a slide-per-patient basis indicates that these models have learned gene expression patterns that encode the spatial locations of cellular distributions.

### 3.3. SPIN-AI Recovers the Spatial Distribution of Gene Expression Clusters

**Figure 3:**
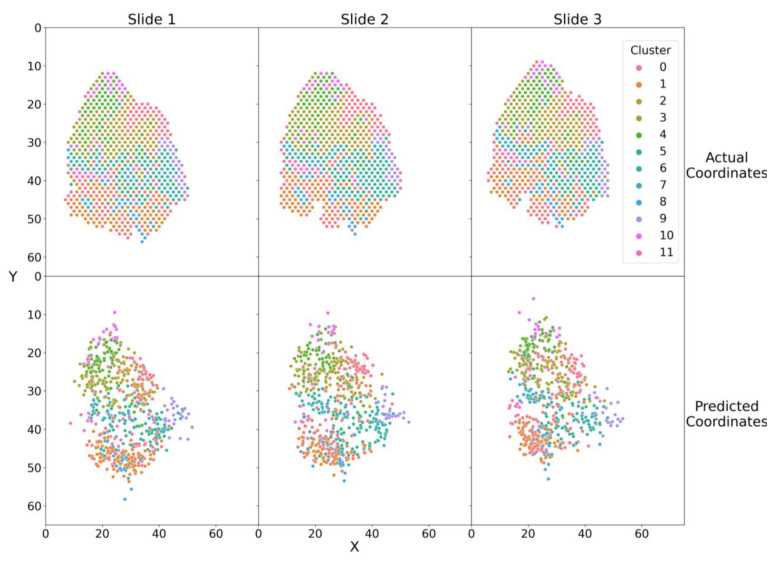
**SPIN-AI encodes spatial gene expression cluster information.** Actual (upper panel) vs. predicted (lower panel) *x* and *y* coordinates of gene expression cluster with respect to each spatial transcriptomic spot on a slide for Patient 2. Spots are colored according to cluster membership as described in Ji et al. [22] See Figure S6 for spatial distribution of gene expression clusters with respect to Patients 5, 9, and 10.

Next, we investigated whether our model could recover the spatial distribution of gene expression clusters as identified using spatial transcriptomics by Ji et al. [22] The total number of gene expression clusters varied among patients (Patient 2, *n* = 11; Patient 5, *n* = 7; Patient 9, *n* = 11; Patient 10, *n* = 6). We used the gene cluster membership to color the spatial spots. The upper panels of Figure 3 and Figure S6 show the actual spatial distribution of spatial spots in tumor slides while the lower panels show their SPIN-AI predicted spatial locations. Our results show SPIN-AI models recover the relative spatial positions of gene expression clusters across slides in each patient, indicating that our models have learned underlying biological signals from the data. Thus, our study illustrated the capability of SPIN-AI models to pick up meaningful spatially distributed gene activities represented as distinct expression clusters.

### 3.4. SPIN-AI Identifies SPGs

We then sought to identify spatially predictive genes (SPGs) whose expression can predict the location of spots on a slide per patient. The procedure for finding SPGs is summarized in Figure S2. Briefly, we used DeepLift to compute gene importance scores, measuring how strongly a gene expression feature contributed to a spot’s prediction. We then averaged individual importance scores across spots in a slide and across slides to achieve an aggregate view of how each gene contributed to the reconstruction of the spatial distribution of the whole tissue sample. SPGs for each patient are listed in Table S2.

We first identified genes with high CSMI scores but also included genes that passed CSMNI and CSPNI criteria. An inspection of CSMI and average expression scores (Figure S3A) showed a non-linear relationship, suggesting that CSMI is biased towards genes that are highly expressed. In turn, genes with larger expressions also possessed more information for encoding spatial representation of cells. Conversely, genes with lower mean expressions also showed spatial expression patterns, and we reason that CSMNI and CSPNI scores aid with the detection of these genes. Figure S3B demonstrates that CSMI underweights genes that have strong importance scores, but only in a limited number of spots. Thus, the inclusion criteria for SPGs use CSMNI and CSPNI to include genes whose contribution is strong and in a reasonably sized subset of spots.

### 3.5. SPGs Recapitulate the Biology of Squamous Carcinoma and Its Cellular Microenvironment

**Figure 4:**
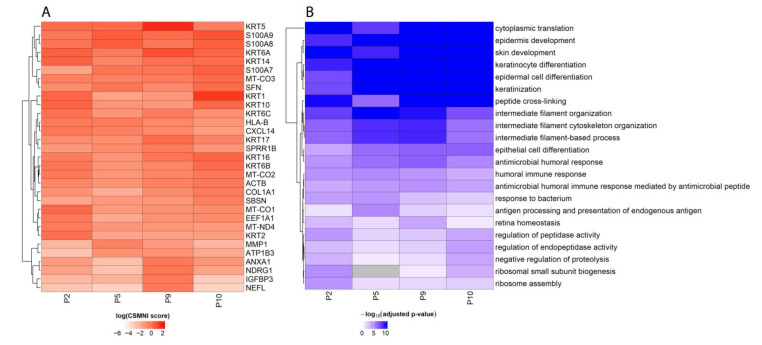
**Top spatially predictive genes and their enriched biological functions.** (**A**) Heatmap showing the union of the top 15 SPGs ranked by CSMNI (cross-slide mean non-zero importance) score per patient (*n* = 31 genes after merging). Heatmap is colored according to log (CSMNI score). (**B**) Heatmap showing union of the top 15 enriched Gene Ontology biological processes of SPGs per patient. Heatmap is colored according to −log10 (adjusted *p*-value).

Figure 4A illustrates the top SPGs (a union of the top 15 SPGs) across each patient ranked by their cross-slide mean non-zero importance, i.e., CSMNI scores. Our results show that these top 15 SPGs are robust across four individual squamous cell carcinoma patients, i.e., most of these SPGs show consistently high importance scores across patients. A substantial number of keratin genes (*KRT5*, *KRT6A*, *KRT6B*, *KRT6C*, etc.) appeared as top SPGs, indicating their roles in forming the cellular matrices that shape the organization of cells in space. Actin-β (*ACTB*) and matrix metallopeptidase 1 (*MMP1*) are also found as top SPGs. The finding of keratin genes, *ACTB*, and *MMP1* as SPGs is not surprising, as they have been known to be involved in cellular organization functions, particularly in squamous cell carcinoma [29,30]. This indicates that our trained SPIN-AI models indeed captured meaningful gene activities that encode cellular organization in local niches. However, the finding of mitochondrial-encoded genes (*MT-CO1*, *MT-CO2*, and *MT-CO3*), which are pertinent to cytochrome-c oxidase activities, and solute carriers (*S100A7*, *S100A8*, *S100A9*, etc.) as top SPGs is unexpected. This indicates unknown spatial-encoding properties of these genes, at least in the case of squamous cell carcinoma, in addition to their known biological functions.

Figure 4B shows the top enriched Gene Ontology biological processes of the SPGs per patient. We show enriched biological processes pertinent to the skin cancer microenvironment, such as the skin’s development of keratinocyte differentiation and an immune response. Our results suggest that SPGs play a role in shaping the microenvironment of squamous cell carcinoma [31,32,33]. Complete enrichment results are listed in Table S3.

Next, we analyzed which SPGs serve as effective tumor markers for different spatial clusters based on their contribution scores (Figure S7, Table S4). We used the tumor-specific keratinocyte (TSK) gene signature derived by Ji et al. [22] and a single-cell RNA-seq to derive the tumor TSK score and further pinpoint the key genes in each cluster. The clusters were annotated with a tumor score, indicating which cluster is enriched for tumor-specific keratinocytes. Few SPGs were commonly shared as cluster markers for the TSK-enriched clusters across the four patients. Patients 2 and 5 shared *MMP1*, *ACTB*, and *TGFB1* while Patients 9 and 10 shared *COL1A1*, *COL1A2*, *HLA-A*, and *HLA-B*. These findings recapitulate the importance of matrix remodeling and immune processes in the development of the squamous cell carcinoma tumor microenvironment while highlighting how these processes uniquely reflect the spatial organization of different patient’s tumors.

**Figure 5:**
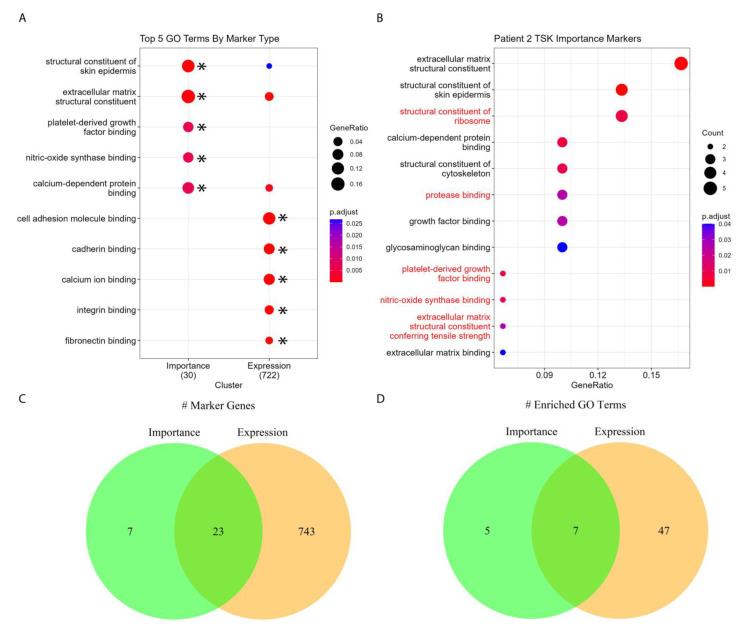
**Comparison of GO terms detected by gene importance score and expression for the top tumor specific keratinocyte (TSK) cluster in Patient 2.** (**A**) Top five GO molecular functions enriched by importance and expression markers. The expression markers overlap with importance markers in enriched terms. Asterisks (*) denote that this term was one of the top five enriched terms for the respective marker set. (**B**) All molecular functions enriched by importance markers. Terms colored black are also enriched by expression markers and terms colored red are uniquely enriched by importance markers. (**C**) Venn diagram comparing the number of marker genes by expression and importance. (**D**) Venn diagram comparing the number of GO molecular functions enriched by expression and importance markers. Count refers to the number of genes from the input gene list associated with the term. Gene ratio is defined as the percentage of input genes associated with an ontology term.

Since Cluster 10 is the most enriched for TSKs in Patient 2, for illustrative purposes, we performed enrichment analyses to determine whether the enriched processes being retrieved from importance scores or gene expression values was pertinent to the biology of squamous cell carcinoma (Table S5). Figure 5A,B show the molecular functions enriched by the importance scores of tumor marker genes compared against those enriched by the gene’s expression scores. We enriched the molecular functions to examine the specific biochemical roles of our marker genes. Enrichment by both importance scores and expression values revealed the involvement of processes such as extracellular matrix binding and structure, cytoskeletal structure, and growth factor binding, which are pertinent to squamous cell carcinoma progression. Further, nitric oxide synthase binding was also enriched, and the role of nitric oxide in the progression of squamous cell carcinoma has been reported [34,35]. Our results show that gene importance scores, which measure a gene’s spatial predictive power, do indeed capture meaningful biological information.

### 3.6. SPGs Discover Unique Biology

**Figure 6:**
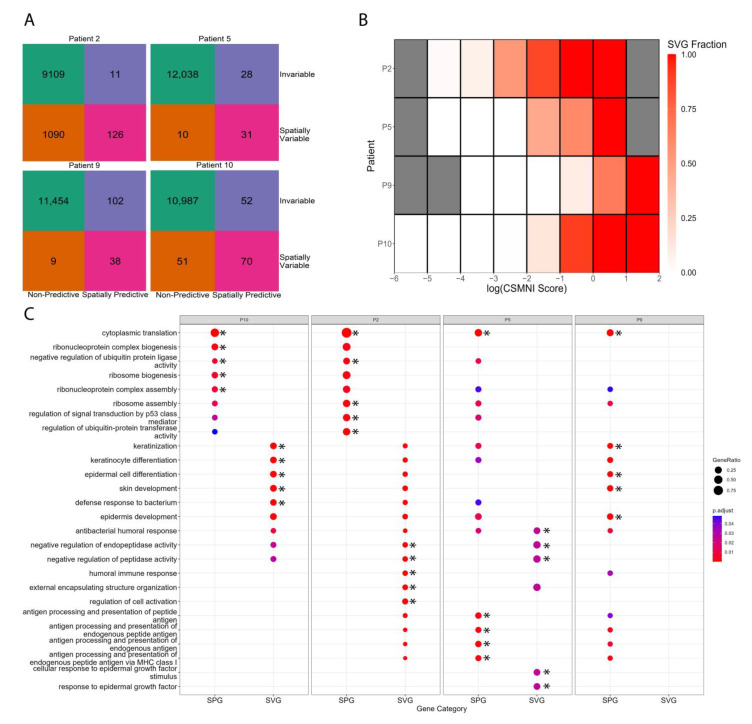
**Identity comparisons between spatially predictive genes (SPGs) and spatially variable genes (SVGs).** (**A**) Number and distribution of SPGs and SVGs across all four patients. (**B**) Fraction of genes within gene importance bins that are SVGs. (**C**). Union of the top five most significant GO biological processes enriched by unique SPGs and SVGs in each patient. Unique SPGs are SPGs that are not SVGs, and unique SVGs are SVGs that are not SPGs. Gene ratio is the proportion of SPGs/SVGs associated with a biological process. For Patient 9, unique SVGs were not enriched in any biological processes. Asterisks (*) denote that this term was one of the top five enriched terms for the respective marker set.

Next, we investigated the overlap between SPGs and SVGs. Figure 6A shows the number of genes that possess spatially predictive and spatially variable properties across four patients. Our results indicate that most genes are neither spatially predictive nor spatially variable (upper left box of Figure 6A). Genes with spatially predictive properties, i.e., SPGs, are those in the lower left and lower right boxes of Figure 6A. Likewise, genes that show spatial variable gene expression, i.e., SVGs, are those in the lower right and upper right boxes of Figure 6A. Genes that are both SPGs and SVGs are located in the lower right boxes of Figure 6A. Our results show that many SPGs are distinct from SVGs, whose expression does not exhibit spatial variability (upper right boxes in Figure 6A), particularly those of Patients 5, 9, and 10. While the heatmap in Figure 6B shows that a large proportion of high CSMNI scoring genes are SVGs, Figure 6A shows that many SVGs are not SPGs in Patients 2 and 10. These results indicate that SPGs are a distinct but overlapping class of genes compared to SVGs.

Since previous analyses showed that a substantial number of the top-ranked SPGs comprise keratin, solute carriers, and mitochondrial-encoded genes (Figure 4A), we sought to investigate how these SPGs are distributed as SVGs and non-SVGs. Intriguingly, a substantial number of SPGs are ribosomal genes (>10 genes, SVG and non-SVG, per patient), many of which are non-SVGs (Figure S8A). In Figure S8B, we illustrate two examples of ribosomal genes that are SPGs, one for which expression is invariable across the slide (RPLP2) and another that is spatially differentially expressed (RPS12), as an illustrative example using Patient 10. On the other hand, keratin, solute carriers, and mitochondrial genes have more equal blends of SVGs and non-SVGs. We then performed a GO-enrichment analysis to understand what biological processes are enriched by the unique SPGs and SVGs (SPG-only and SVG-only genes, respectively, Table S6). Unique SPGs were unexpectedly enriched in their ribosomal functions across all four patients (Figure 6C). Although altered ribosome biogenesis is known to play a pivotal role in tumorigenesis [36,37] and ribosome composition can fluctuate to regulate protein synthesis for specialized functions [38,39], the spatial modulatory roles of ribosomal genes in determining the properties of the tumor microenvironment are presently not known. Recent evidence suggesting the tissue-specific expression of ribosomal genes [40], stoichiometric regulation of ribosomal proteins [41], and context-dependent specialization of ribosomal activities [42] may explain the spatial-regulatory roles of ribosomal genes. As ribosome assembly exhibits context-dependency and ribosomal genes tend to be SPGs and not SVGs, our results suggest that the interaction between ribosomal genes contributes to the determination of the spatial architecture of squamous cell carcinoma.

In the cases of Patients 5 and 9, antigen-processing functions were also enriched alongside ribosomal processes (Figure 6C), matching the patterns in Figure 4B in which cytoplasmic translation was enriched by SPGs across all patients while antigen processing was primarily enriched in Patients 5 and 9, suggesting that these two patients’ tumors had unique immune profiles. Unique SVGs were primarily enriched in skin differentiation and peptidase activity regulation, which were common functions of many SPGs (Figure 4B). Taken together, our results suggest that not only do SPGs capture similar information to SVGs, but they can also capture unique biological contributors to spatial architecture.

## 4. Discussion

Technological advances in spatially resolved expression profiling [13] via imaging mass cytometry [43,44], multiplexed ion beam imaging [45], and spatial transcriptomics have opened new avenues for understanding spatial cellular distribution. Such data resolutions allow for the deeper exploration of genes and the associated molecular processes that modulate spatial architecture and cell–cell communication [46].

A number of computational approaches have been developed in recent years to integrate single-cell and spatial transcriptomic data [47]. This includes methods such as Seurat [23], SpaGE [48], and LIGER [49]. Beyond data integration, additional computational methods such as trendsceek [18], SpatialDE [19], SPARK [20], and SOMDE [21] have been developed to identify spatially variable genes (SVGs), whose expression patterns are statistically distinct in space. The detection of SVGs is, in principle, similar to the identification of differentially expressed genes in bulk RNA-seq (DEGs). Like DEGs, which reveal which genes and associated pathways are activated or inactivated in comparison to a particular biological state, SVGs can inform on genes and corresponding pathways that show spatial activity variations in a tissue or cellular microenvironment.

Another line of effort focuses on building computational methods for reconstructing cellular spatial organization using only single-cell transcriptomic data as an input [50]. These methods rely on certain educated assumptions, such as physically adjacent cells tending to share similar gene expression profiles [17] and cellular spatial organization being in part able to be recapitulated by local ligand–receptor interaction profiles [51]. Although these assumptions have advantages, they do not fully answer “how cells know where they are” [52]. Nevertheless, these methods show that single-cell transcriptomic data indeed encode information about the spatial organization of cells in a biological niche.

Based on these observations, we therefore hypothesize that expression profiles of certain genes carry information regarding the spatial organization of cells within tissues. We termed such genes as spatially predictive genes (SPGs). However, unlike SVGs, SPGs do not necessarily show expression variations in space, i.e., the expression of some SPGs can be broadly distributed across regions in cellular niches and do not necessarily show similar expression in adjacent cells. Rather, SPGs demonstrate predictive power in predicting spatial locations.

We therefore sought to devise a totally unbiased strategy, i.e., without pre-defined assumptions on spatial differential expression, for detecting SPGs using artificial intelligence (AI) methods, particularly deep learning approaches. A number of artificial intelligence (AI) methods involving deep learning have been applied to spatial transcriptomic data [53], including SVG detection [21,54], cluster analysis [55,56,57], cell communication analysis [58,59], and data imputation [60,61,62,63]. However, to our knowledge, no AI method has yet been developed to detect SPGs.

In this work, we developed Spatially Informed AI (SPIN-AI), an AI platform consisting of a deep, feedforward neural network that aims to predict the spatial locations, i.e., the *x* and *y* coordinates, of a spot derived from 2D images of single-cell transcriptomic slides. As deep neural networks possess the capability to detect nonlinear interactions between input features, we reasoned that deep neural networks can better capture cooperative signals from gene activities that relate to spatial location than conventional SVG detection methods that test a single gene at a time. The ability of trained deep learning models to predict the location of spatial transcriptomics would indicate that the model has learned the identity of genes whose expression profiles are predictive of cell organization in space. Here, we used spatial transcriptomic data obtained from squamous cell carcinoma [22] as a proof-of-concept study. In particular, we devised gene importance scores to capture the overall importance of a gene in predicting spatial locations of all spots in a slide image and derived SPG candidates from these importance scores.

We showed that our trained deep learning model could predict spot locations in all three slides for each patient, indicating that the model indeed captures gene expression signals that are spatially predictive (Figure 2 and Figure 3). We demonstrated that top-scored SPGs recapitulated the biology of squamous cell carcinoma, including in skin development and humoral immune responses (Figure 4 and Figure 5). Our results suggest that genes that play important roles in tumorigenesis are not just involved in disease etiology but also play key roles in remodeling the cellular niche and determining spatial cellular organization. Next, we illustrated that SPGs are indeed a new class of genes that are distinct from SVGs (Figure 6). In particular, we found a substantial number of ribosomal genes were SPGs, whose expression profiles predicted spot location but were not differentially expressed in space (i.e., not spatially variable). The discovery of ribosomal genes acting as SPGs was unexpected; although they are known to play key roles in regulating protein synthesis in tumor cells [64,65], their spatial-regulating roles in shaping cell organization in a disease niche are not yet known.

While SPIN-AI can detect biologically informative SPGs, this pipeline can be improved in several ways to help with the identification of SPGs. The CSMI threshold for determining SPGs could be identified through a more data-driven process, and further exploration of how threshold choice influences SPG identification is needed. The threshold is currently programmed as a user-defined parameter to allow users to adjust it to their needs. Additionally, the use of importance scores in determining SPGs could be bypassed via feature selection schemes, such as recursive feature elimination or relief feature selection. By determining a smaller set of features that maintain or improve model performance, these features may comprise a better-defined set of SPGs than those selected based on importance scores. However, the lack of a ground-truth set of SPGs makes this comparison difficult.

Nevertheless, we have shown that SPIN-AI is an AI platform capable of detecting SPGs that can be applied to a variety of biological conditions. We also anticipate that the identification of SPGs could shed new light on spatial analyses and deepen our understanding of how the activities of genes determine cell organization and shape their microenvironment to sustain cellular phenotypes in diseased tissue.

## Data Availability

The code, scripts, and resources can be found at github: https://github.com/HuLiLab/SPIN-AI (accessed on 17 May 2023).

## Supplementary Materials

The following supporting information can be downloaded at: https://www.mdpi.com/article/10.3390/biom13060895/s1. Figure S1. Flowchart outlining key analysis steps and code package usage. Figure S2. Flowchart for determining spatially predictive genes (SPGs) in a single patient. Figure S3. SPIN-AI importance scores. Figure S4. Comparing mean versus median for importance scoring. Figure S5. SPIN-AI cross-slide predictive performance. Figure S6. Spatial distribution of gene expression clusters for Patients 5, 9, and 10. Figure S7. SPG markers and tumor scores in each patient. Figure S8. Distribution of SPG genes per gene category. Table S1. Brief description of which R and Python packages were used in this study. Table S2. SPIN-AI gene annotations per patient. Genes are annotated according to whether they are SPGs and according to their spatial variability, as determined using SpatialDE. Genes are annotated with CSMI, CSMNI, and CSPNI as well as q-values, computed using SpatialDE. Table S3. GO-enrichment analysis results for SPGs per patient. Table S4. Gene importance markers for spatial clusters per patient. Table S5. GO-enrichment analysis results for importance and expression marker genes for Patient 2’s TSK cluster. Table S6. GO-enrichment analysis results for unique SPGs and SVGs across all four patients.
