# Supplementary material for: SPIN-AI: A Deep Learning Model That Identifies Spatially Predictive Genes
Source: Biomolecules. 2023 May 27;13(6):895. doi: 10.3390/biom13060895 (PMC10296445; doi:10.3390/biom13060895)
Supplement: Supplementary file 1 [file biomolecules-13-00895-s001.zip › Supplementary_Data_Figures/Supplementary Figures JPG/Supplementary Materials_Figure_and_Table_Legends.docx]

**Supplementary Materials:**

**Supplementary Figure Legends:**

**Figure S1. Flowchart outlining analysis steps and code package usage.** An outline of the analysis steps and code packages involved at each step. Blue boxes indicate steps done in R, yellow boxes indicate steps done in python. Boxes on the left (lighter colors) indicate preprocessing/execution of the SPIN-AI pipeline. Boxes on the right (darker colors) indicate analysis of SPIN-AI outputs.

**Figure S2**. **Flowchart for determining spatially predictive genes (SPGs) in a single patient**. (**A**) Gene importance scores are computed for each test-fold spot in each cross-validation fold. Here, rows represent spots and k=4 for illustration. Cell colors represent importance scores. (**B**) Cross validation fold results are concatenated together. (**C**) Mean importance, mean non-zero importance, and percentage of non-zero contribution are computed per gene. (**D**) This process is repeated for all 3 slides for each patient. (**E**) Gene-wise statistics for all slides are averaged together and genes are ranked. Genes with the cross-slide mean importance scores above a defined threshold (CSMI; top 3 shown here for illustration purpose) or a cross-slide mean non-zero importance (CSMNI) score larger than the minimum of the top set genes and cross-slide percentage of non-zero importance (CSPNI) scores in more than 20% of spots are considered spatially predictive genes (SPGs). Genes 1-3 have the 3 largest CSMI. Genes 4 and 6 have a CSMNI greater than the minimum for genes 1-3. However, gene 6 has a low CSPNI. Genes 5, 7 and 8 have small CSMI scores and their CSMNI is below the minimum for genes 1-3. (**F**) The final set of spatially predictive genes selected according to our selection criteria.

**Figure S3.** **SPIN-AI** **importance scores.** (**A**) Relationship between gene expression and importance scores in each patient slide. (**B**) CSMI, CSMNI, and CSPNI scores and visualizations of example SPGs from patient 2.

**Figure S4. Comparing mean versus median for importance scoring.** (**A**) Comparing mean importance scores versus median importance scores (top row) and mean non-zero importance scores versus median non-zero importance score (bottom row) across all genes and all slides for each patient. The Pearson correlation between these scores and the patient numbers are shown. (**B**) Comparing mean versus median for cross-slide aggregation for mean importance scores (first row), mean non-zero importance score (second row). For example, the most upper left panel compares the cross-slide average of mean importance scores against the cross-slide median of mean importance scores. The Pearson correlation between these scores and the patient numbers are shown.

**Figure S5. SPIN-AI cross-slide Predictive Performance.** For each patient, a model was fitted on a slide using hyperparameters determined from the cross-validation test and other slides were held out for validation and testing. The slides were arranged in the following order of (training, validation, testing): (slide 1, slide 2, slide3), (slide 2, slide 3, slide 1), (slide 3, slide 1, slide 2). Predictions for the test slides are shown with spots in their original position and colored by their distance between their original and predicted locations. The mean distance error (MDE) was computed for each test slide and shown above.

**Figure S6**. **Spatial distribution of gene expression clusters for patients 5, 9, and 10**. Actual (upper panel) versus predicted (lower panel) *x* and *y* coordinates of gene expression cluster with respect to each spot on a slide per patient are shown.

**Figure S7**. **SPG markers and tumor scores in each patient**. Heatmaps showing statistically significant SPGs and their associated expression clusters with tumor (TSK) score in each patient. Tumor score refers to how strongly a gene expression cluster expresses a tumor-specific keratinocyte gene set derived from Ji et al.[22].

**Figure S8**. **Distribution of SPG genes per gene category**. **A.** The number of spatially variable genes (SVGs) and non-SVGs in ribosomal genes, keratins, solute carriers, and mitochondrial-encoding SPGs. **B.** Examples of spatially variable and invariable ribosomal SPGs from patient 10.

**Supplementary Data:**

**Table S1.** Software package usage and references.

**Table S2.** SPIN-AI gene annotations per patient. Genes are annotated by whether they are SPGs and by their spatial variability as determined by SpatialDE. Genes are annotated with CSMI, CSMNI, and CSPNI as well as q-values computed by SpatialDE.

**Table S3.** GO enrichment analysis results for SPGs per patient.

**Table S4.** Gene importance markers for spatial clusters per patient.

**Table S5.** GO enrichment analysis results for importance and expression marker genes for patient 2’s TSK cluster.

**Table S6.** GO enrichment analysis results for unique SPGs and SVGs across all 4 patients.
