# Supplementary figures and images for: SPIN-AI: A Deep Learning Model That Identifies Spatially Predictive Genes
Source: Biomolecules. 2023 May 27;13(6):895. doi: 10.3390/biom13060895 (PMC10296445; doi:10.3390/biom13060895)

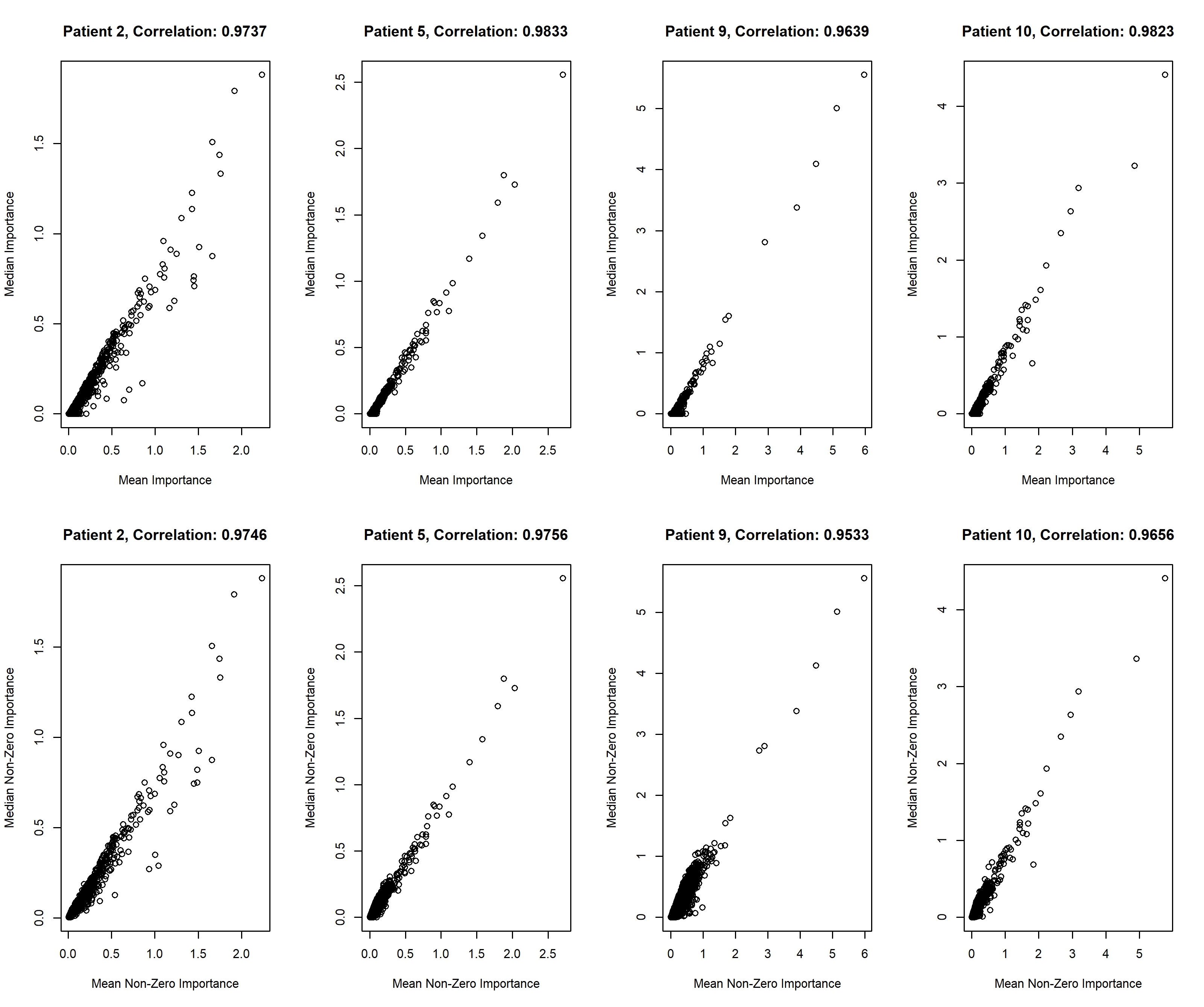

Supplement: Supplementary file 1 [file biomolecules-13-00895-s001.zip › Supplementary_Data_Figures/Supplementary Figures JPG/Supplementary_Figure_4a.jpg]

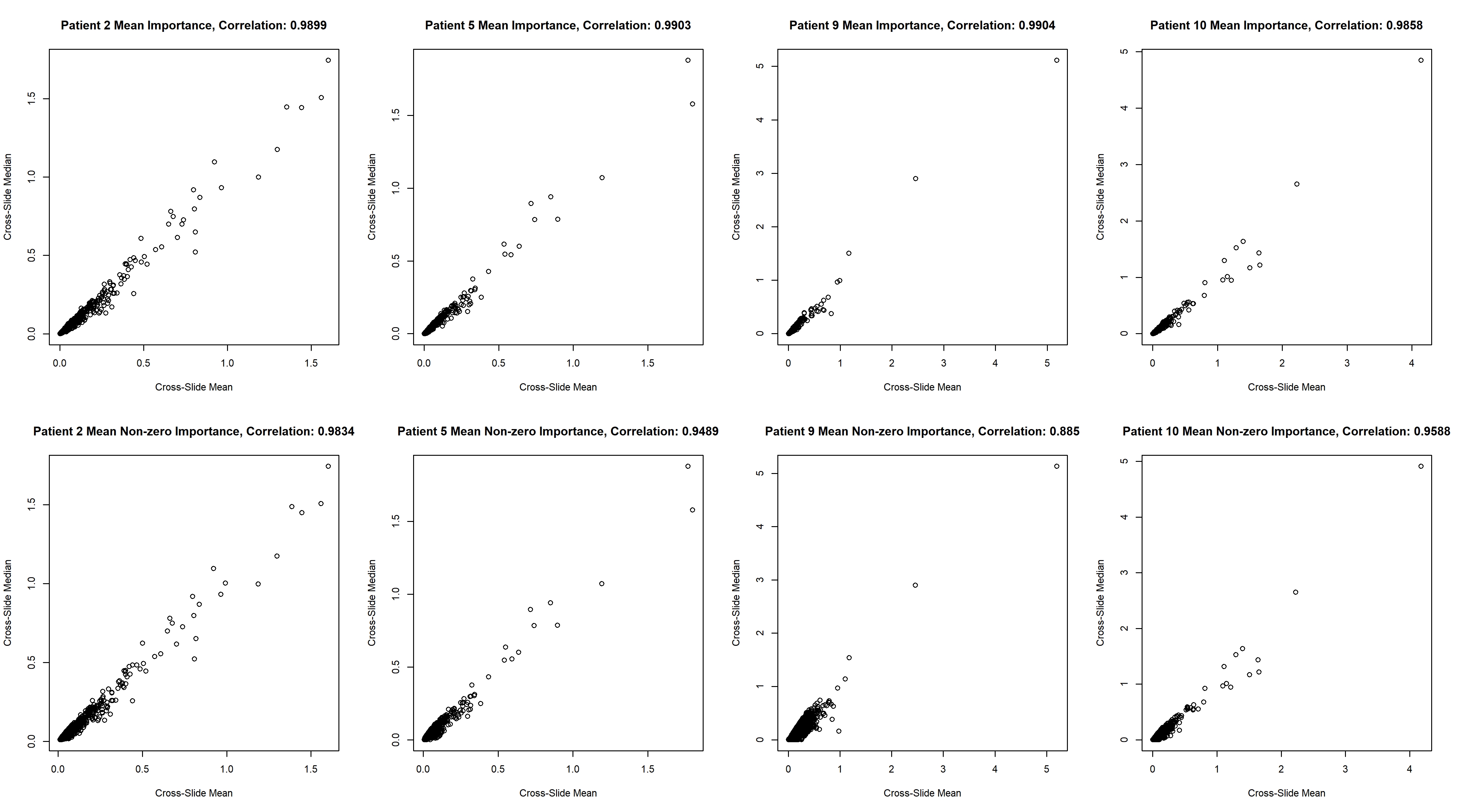

Supplement: Supplementary file 1 [file biomolecules-13-00895-s001.zip › Supplementary_Data_Figures/Supplementary Figures JPG/Supplementary_Figure_4b.jpg]

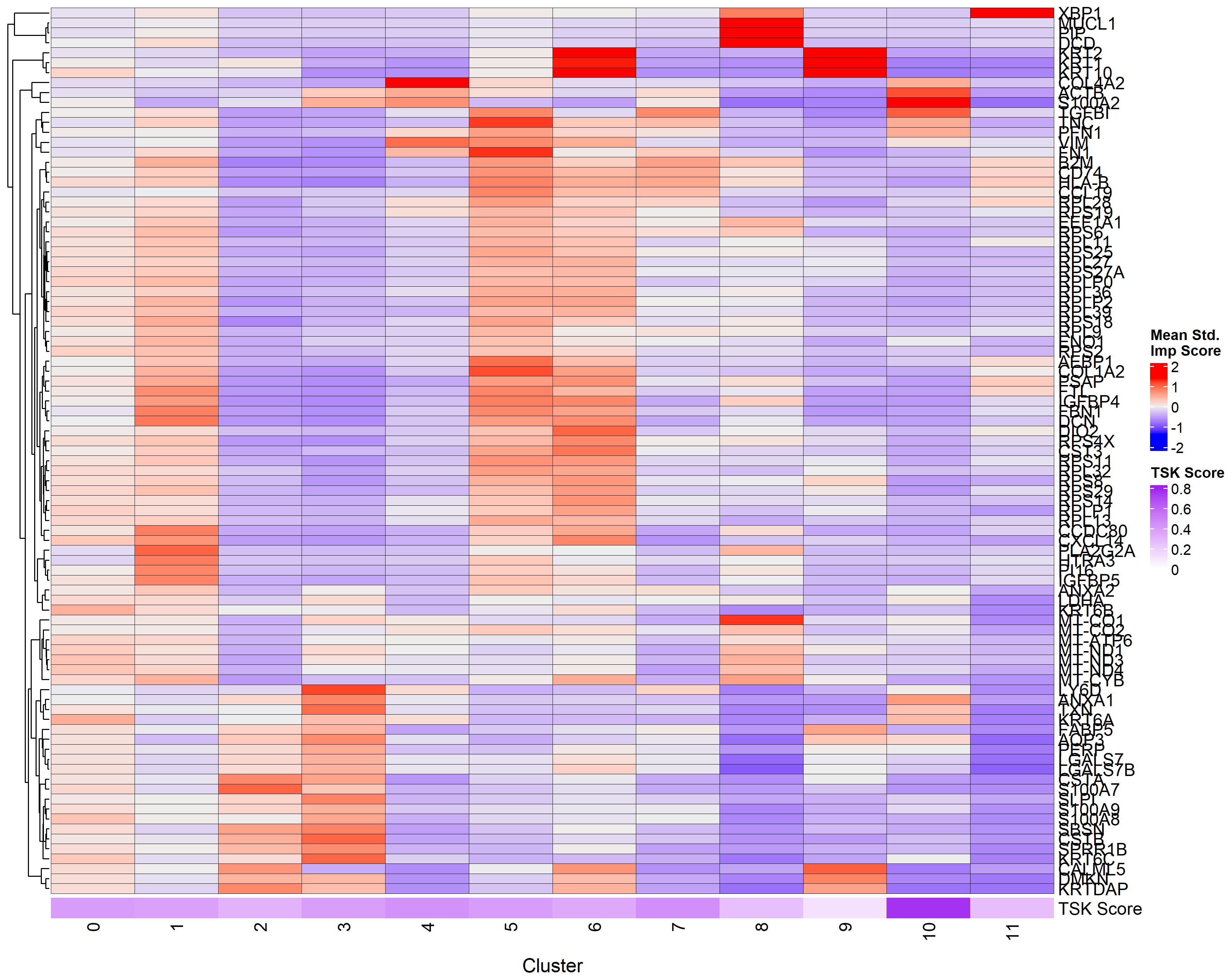

Supplement: Supplementary file 1 [file biomolecules-13-00895-s001.zip › Supplementary_Data_Figures/Supplementary Figures JPG/P2_Supplementary_Figure_7.jpg]

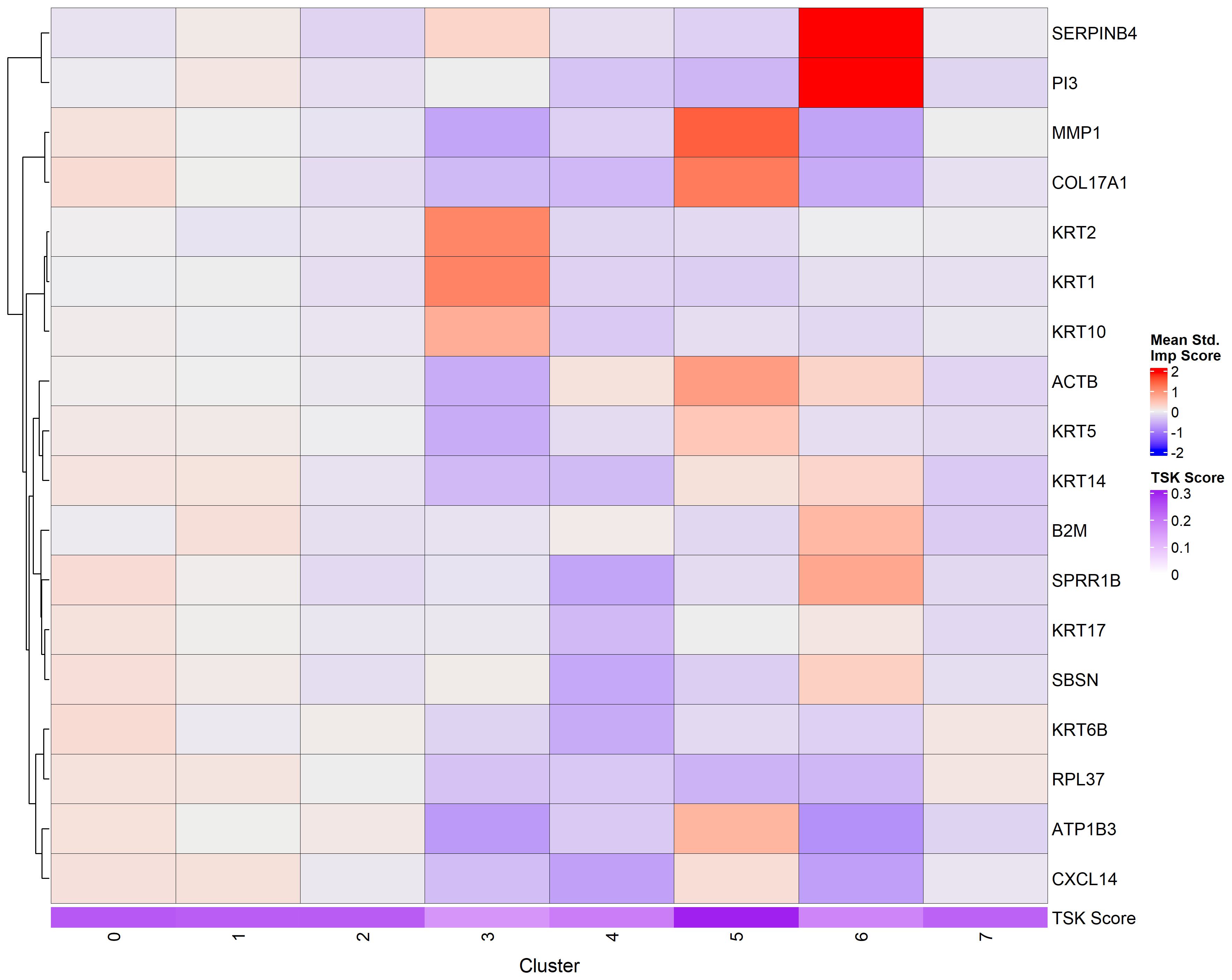

Supplement: Supplementary file 1 [file biomolecules-13-00895-s001.zip › Supplementary_Data_Figures/Supplementary Figures JPG/P5_Supplementary_Figure_7.jpg]

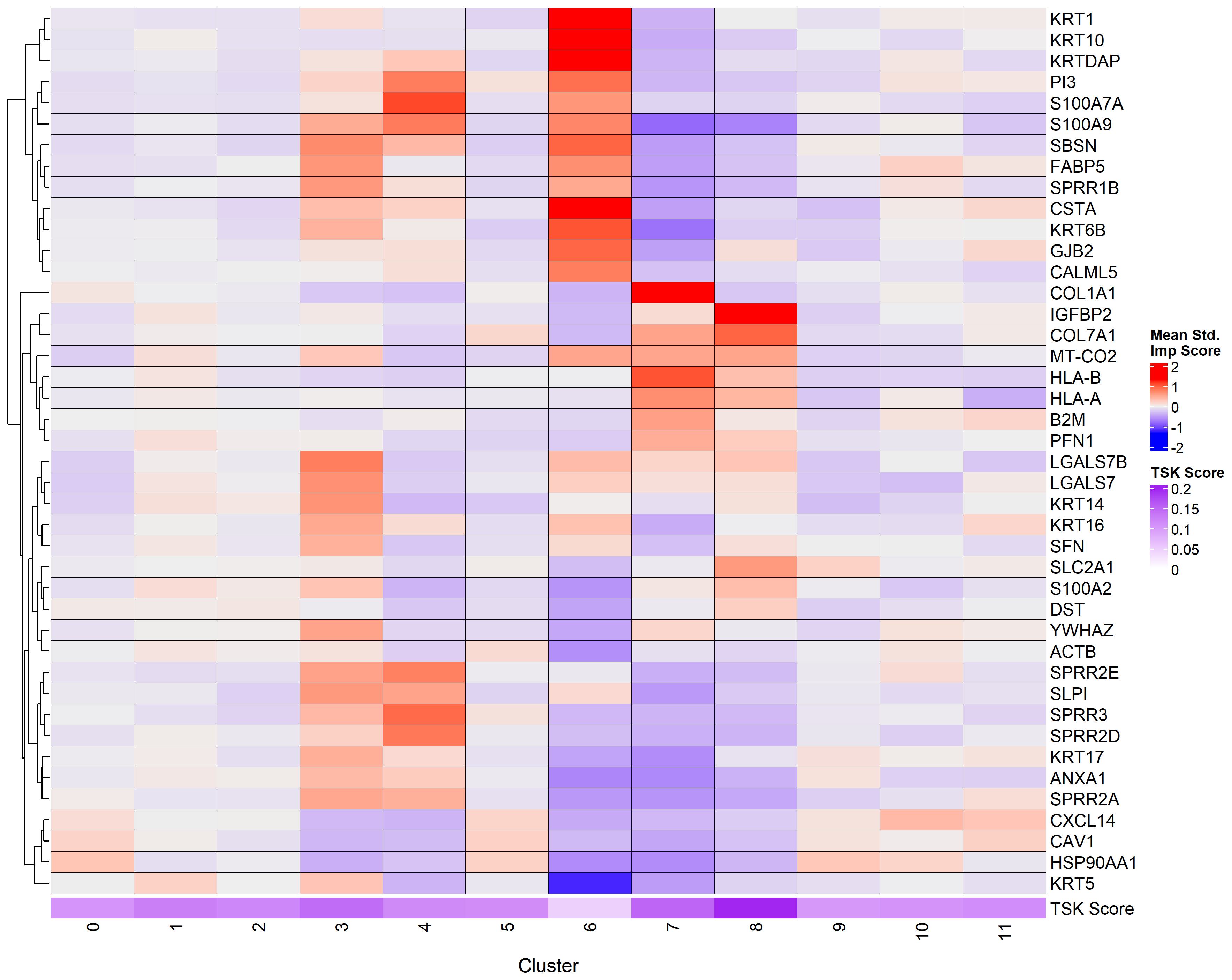

Supplement: Supplementary file 1 [file biomolecules-13-00895-s001.zip › Supplementary_Data_Figures/Supplementary Figures JPG/P9_Supplementary_Figure_7.jpg]

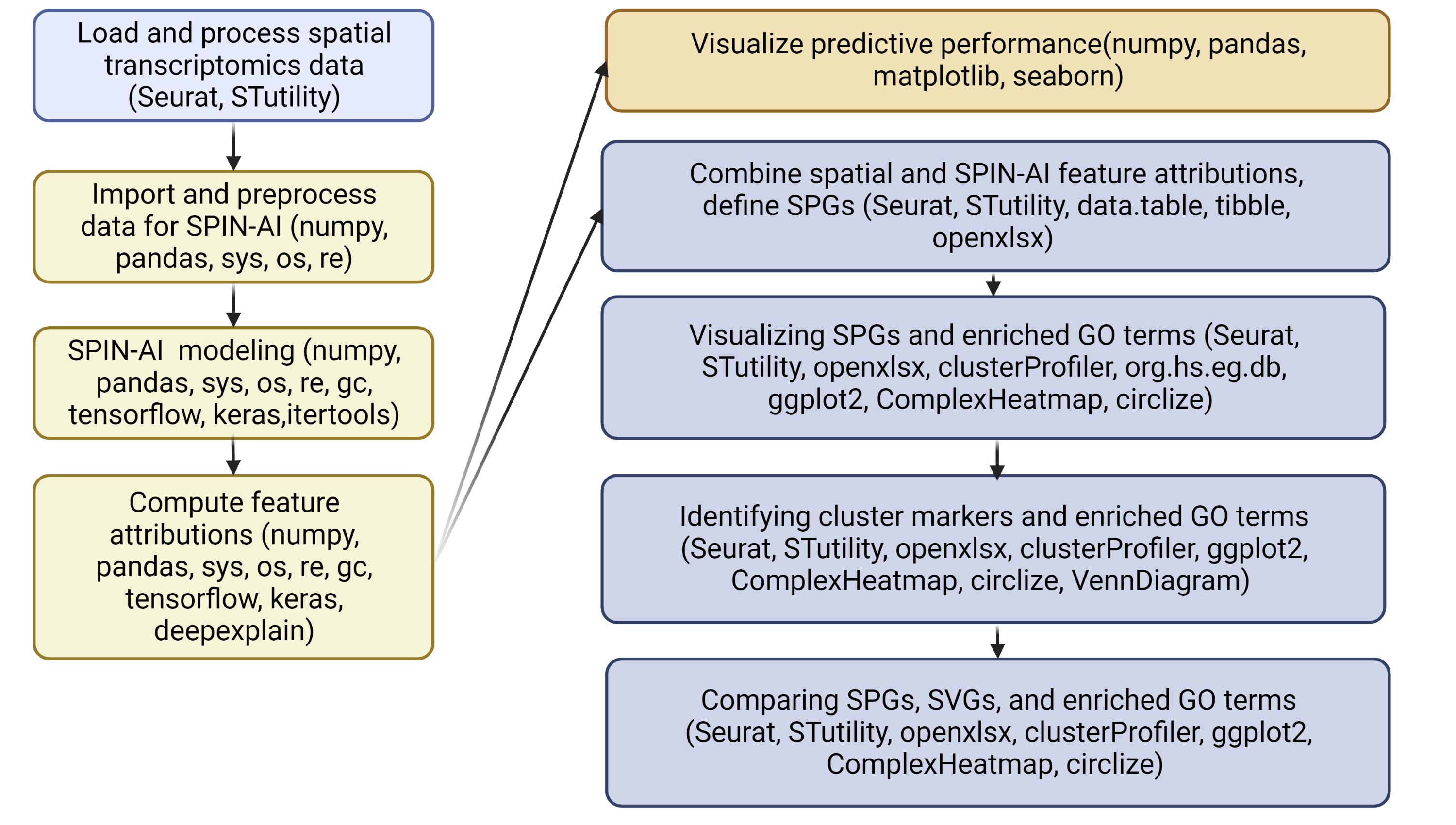

Supplement: Supplementary file 1 [file biomolecules-13-00895-s001.zip › Supplementary_Data_Figures/Supplementary Figures JPG/Supplementary_Figure_1.jpg]

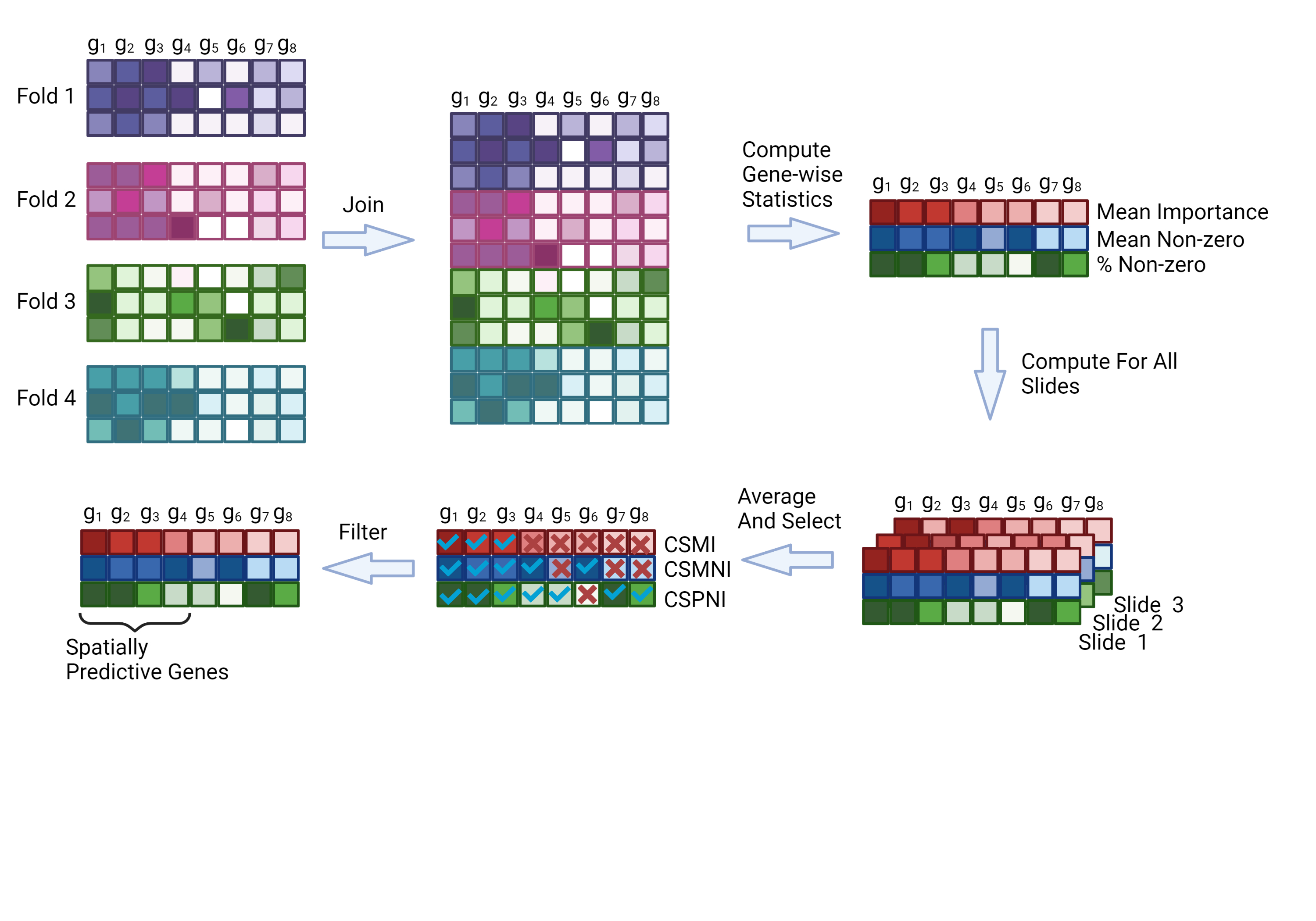

Supplement: Supplementary file 1 [file biomolecules-13-00895-s001.zip › Supplementary_Data_Figures/Supplementary Figures JPG/Supplementary_Figure_2.jpg]

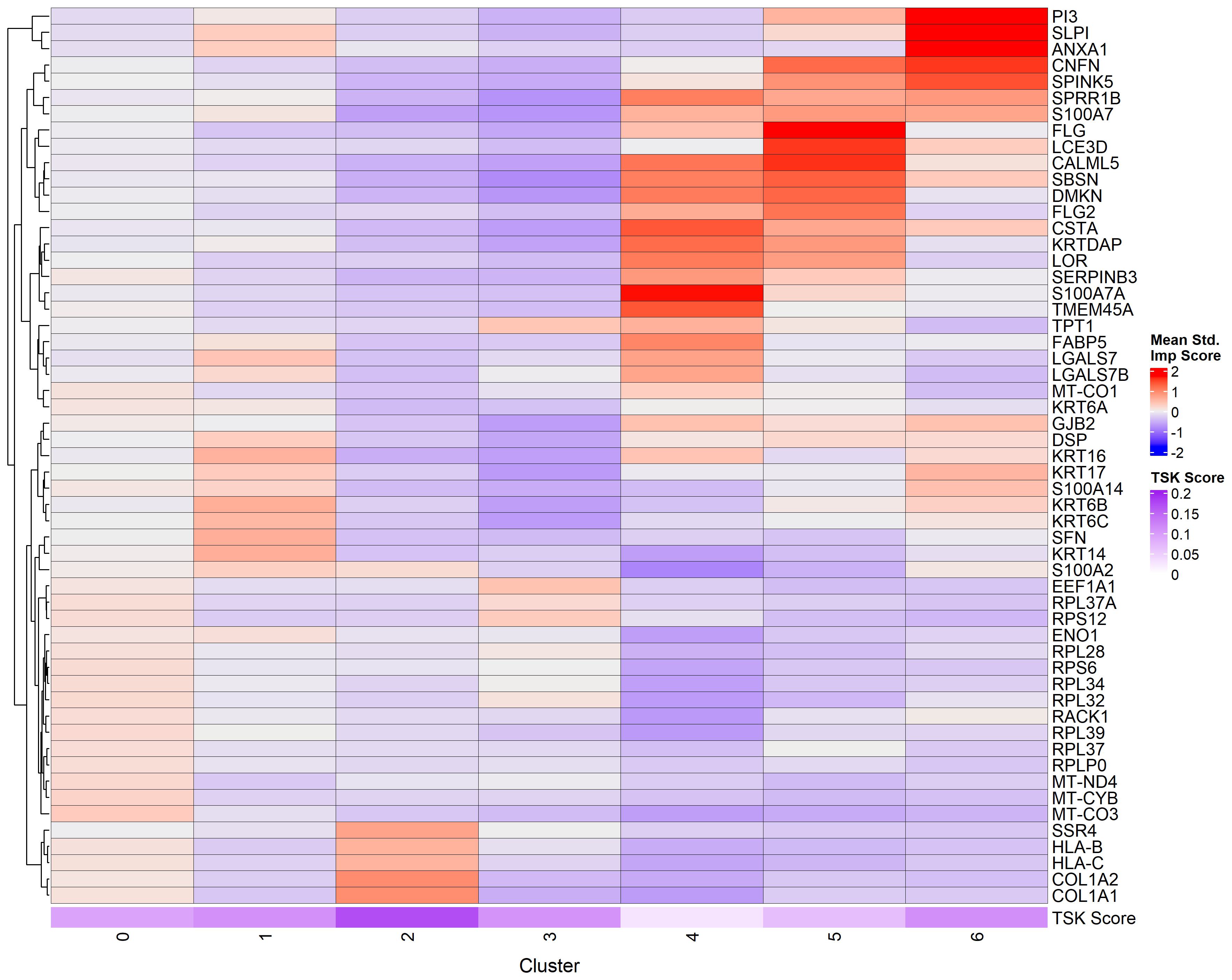

Supplement: Supplementary file 1 [file biomolecules-13-00895-s001.zip › Supplementary_Data_Figures/Supplementary Figures JPG/P10_Supplementary_Figure_7.jpg]
